# Supplementary figures and images for: Temporal Dynamics of the Gut Bacteriome and Mycobiome in the Weanling Pig
Source: Microorganisms. 2020 Jun 9;8(6):868. doi: 10.3390/microorganisms8060868 (PMC7356342; doi:10.3390/microorganisms8060868)

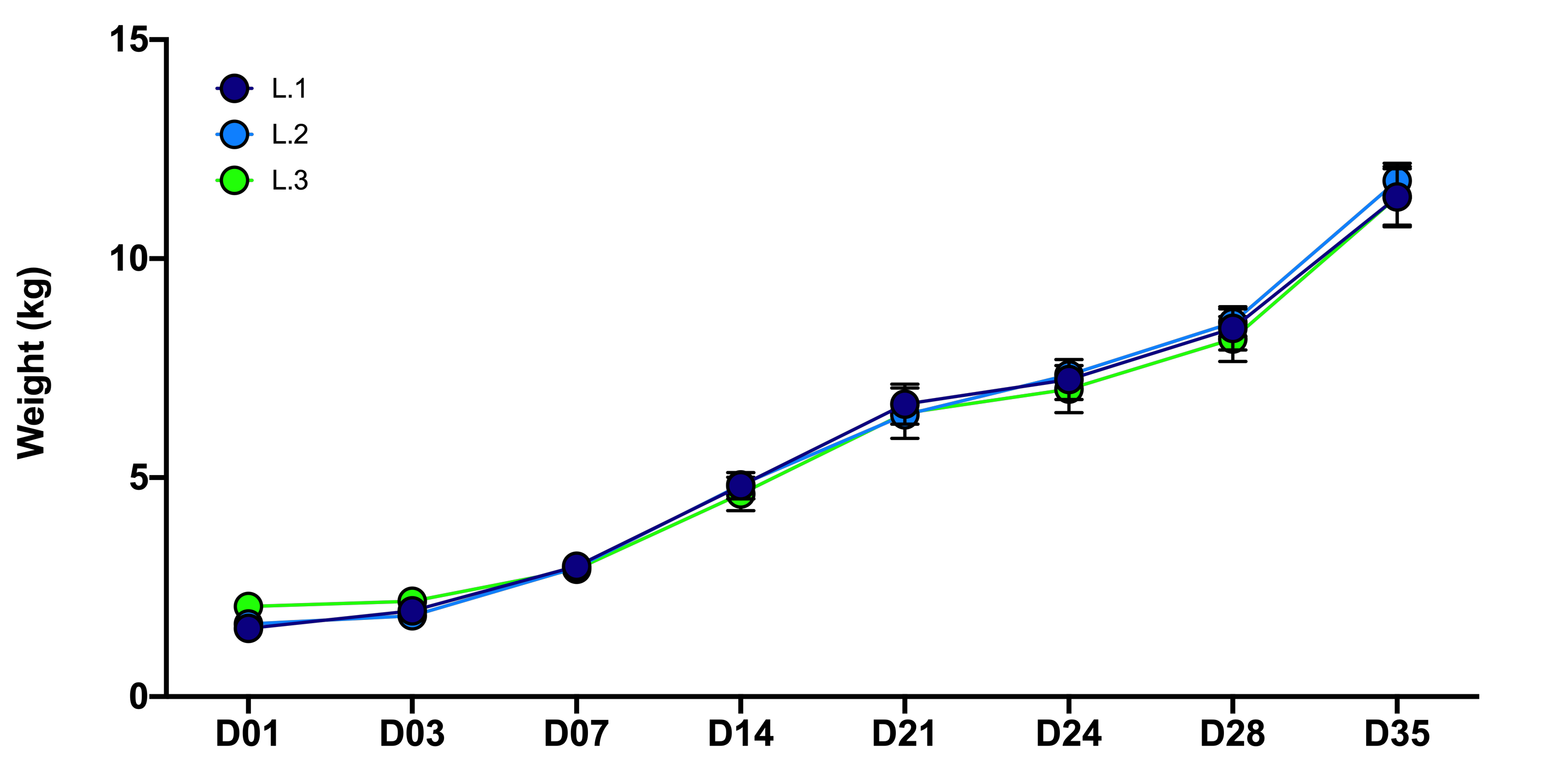

Supplement: Supplementary file 1 [file microorganisms-08-00868-s001.zip › Temporal Dynamics Supplemental Figures/SI_Fig1.tiff]

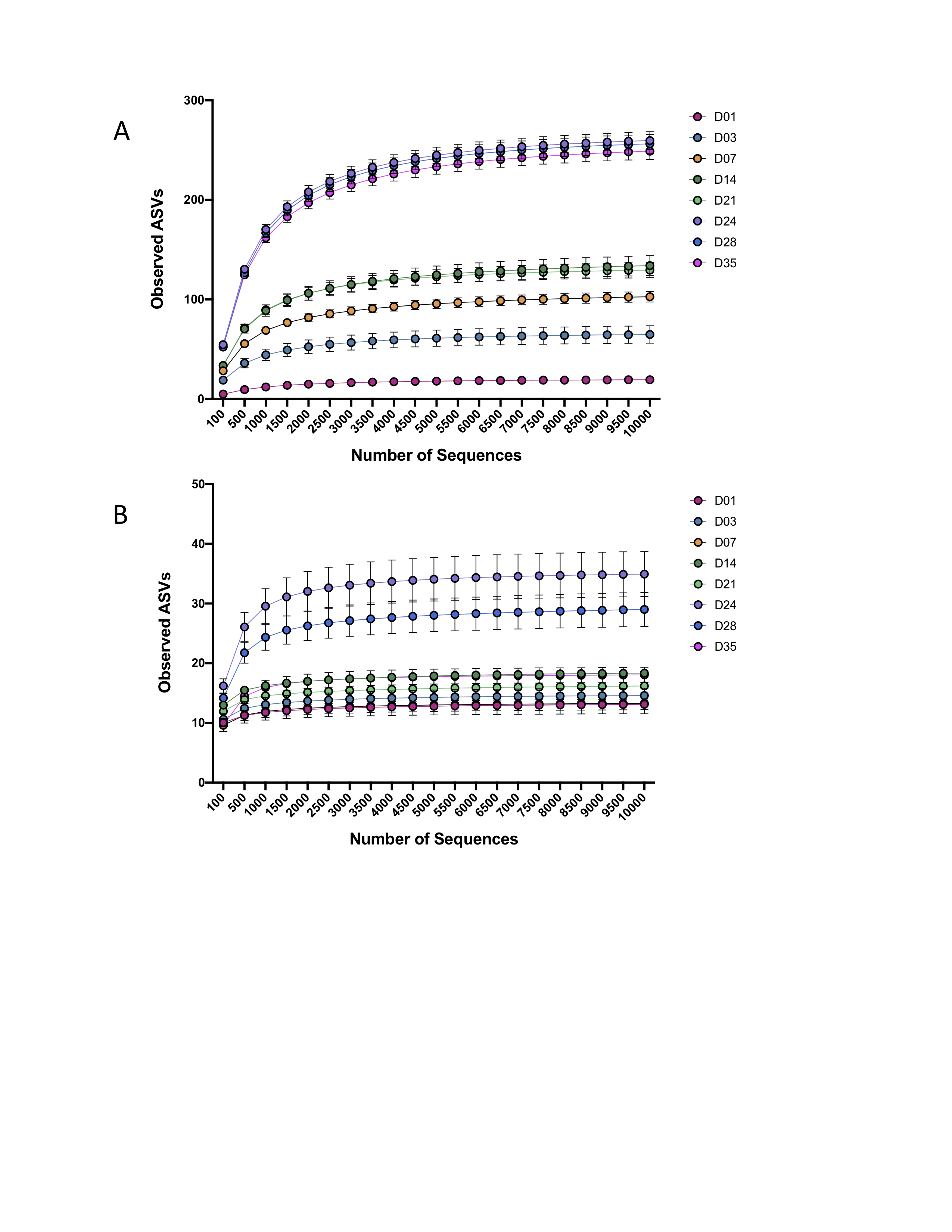

Supplement: Supplementary file 1 [file microorganisms-08-00868-s001.zip › Temporal Dynamics Supplemental Figures/SI_Fig2.tiff]

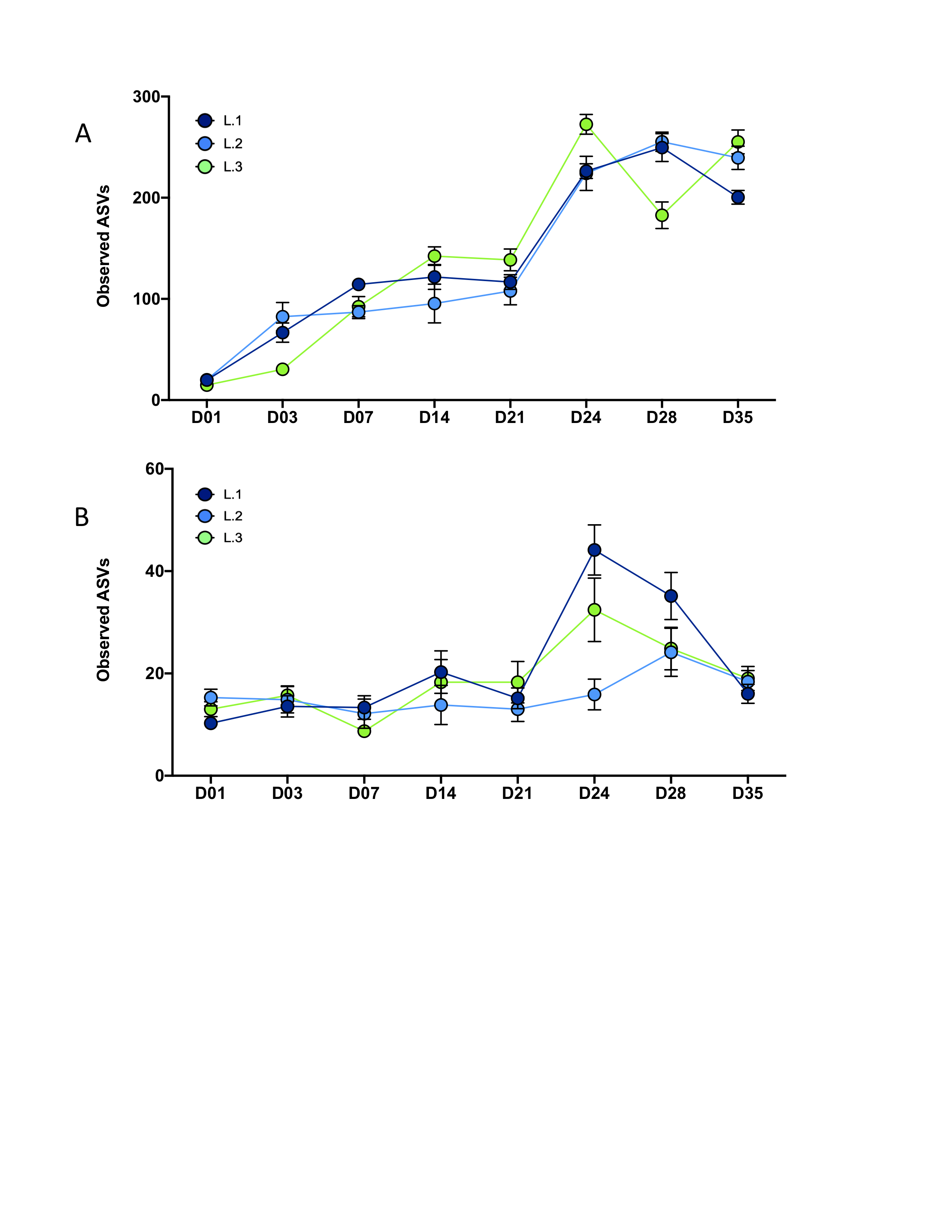

Supplement: Supplementary file 1 [file microorganisms-08-00868-s001.zip › Temporal Dynamics Supplemental Figures/SI_Fig3.tiff]

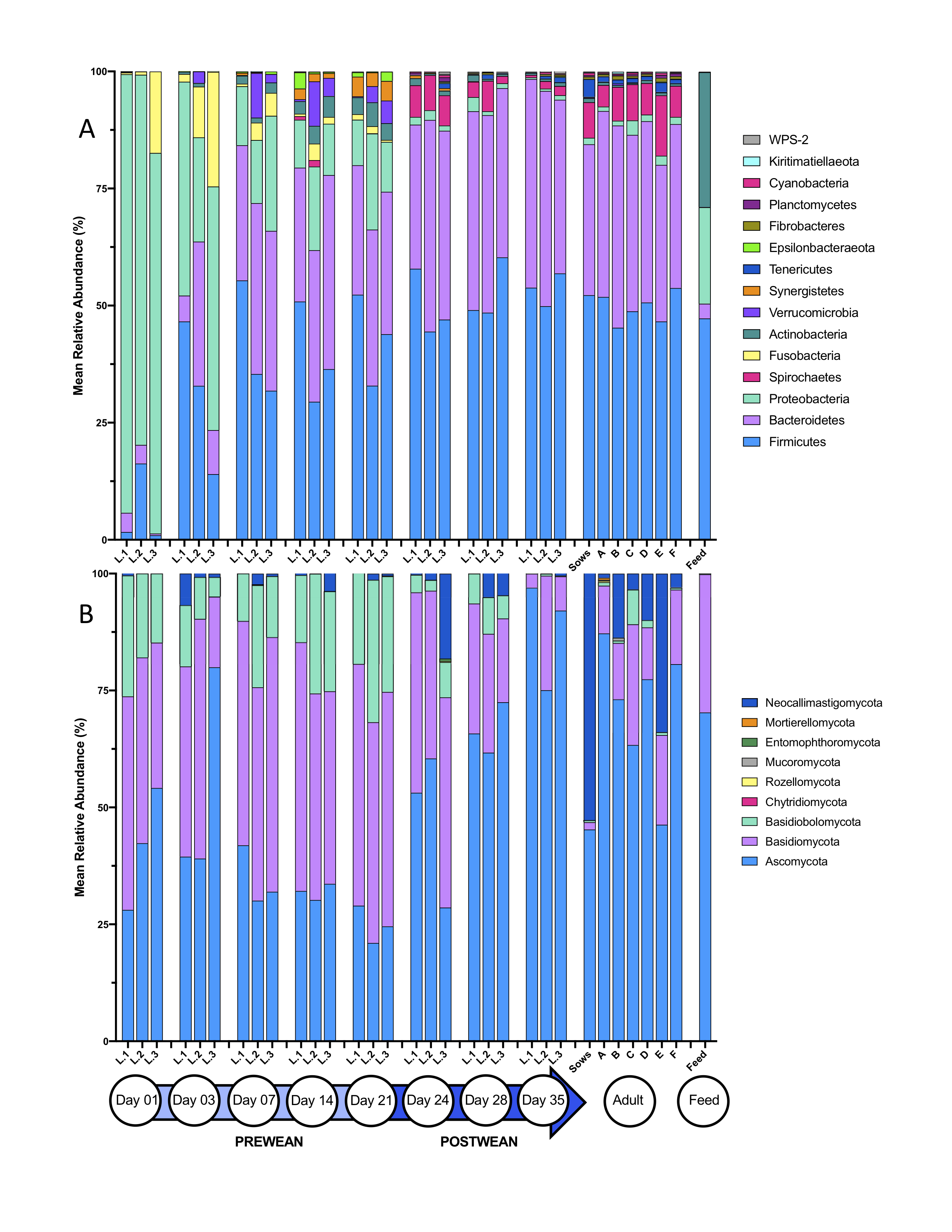

Supplement: Supplementary file 1 [file microorganisms-08-00868-s001.zip › Temporal Dynamics Supplemental Figures/SI_Fig4.tiff]

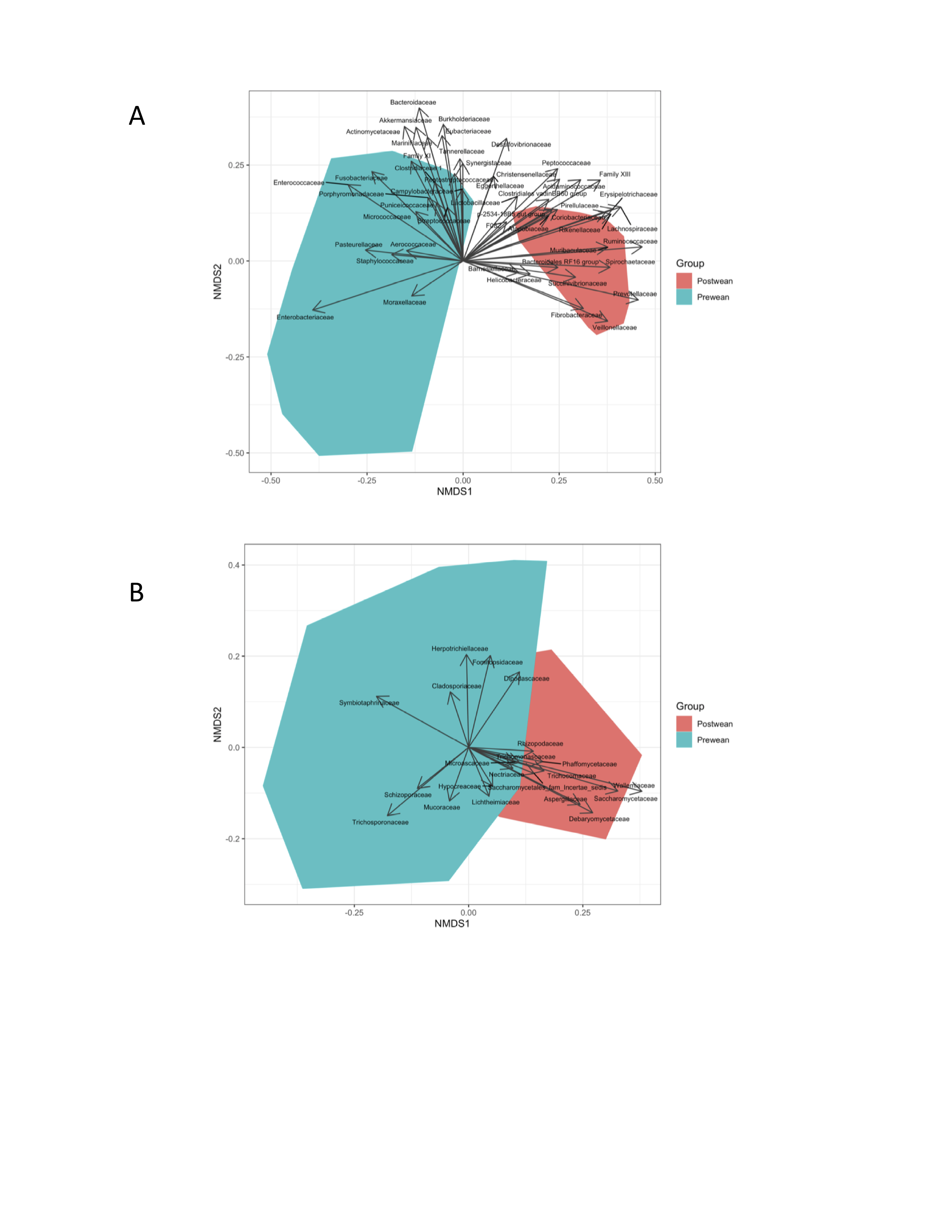

Supplement: Supplementary file 1 [file microorganisms-08-00868-s001.zip › Temporal Dynamics Supplemental Figures/SI_Fig5.tiff]
